# Effects of Sitagliptin Treatment on Dysmetabolism, Inflammation, and Oxidative Stress in an Animal Model of Type 2 Diabetes (ZDF Rat)

**DOI:** 10.1155/2010/592760

**Published:** 2010-06-21

**Authors:** Liliana Ferreira, Edite Teixeira-de-Lemos, Filipa Pinto, Belmiro Parada, Cristina Mega, Helena Vala, Rui Pinto, Patrícia Garrido, José Sereno, Rosa Fernandes, Paulo Santos, Isabel Velada, Andreia Melo, Sara Nunes, Frederico Teixeira, Flávio Reis

**Affiliations:** ^1^Institute of Pharmacology & Experimental Therapeutics, IBILI, Medicine Faculty, University of Coimbra, 3000-354 Coimbra, Portugal; ^2^ESAV, Polytechnic Institute of Viseu, 3500 Viseu, Portugal; ^3^Pharmacology & Pharmacotoxicology Unit, Faculty of Pharmacy, Lisbon University, 1649-003 Lisboa, Portugal; ^4^Functional Genomics Laboratory, Center of Histocompatibility of the Centre, 3001-301 Coimbra, Portugal; ^5^Institute for Molecular and Cellular Biology, Porto University, 4150 Porto, Portugal

## Abstract

The purpose of this paper is to evaluate the chronic effect of sitagliptin on metabolic profile, inflammation, and redox status in the Zucker Diabetic Fatty (ZDF) rat, an animal model of obese type 2 diabetes. Diabetic and obese ZDF (*fa/fa*) rats and their controls (ZDF +/+) were treated during 6 weeks with vehicle (control) and sitagliptin (10 mg/kg/bw). Glucose, HbA1c, insulin, Total-c, TGs, IL-1*β*, TNF-*α*, CRP*hs*, and adiponectin were assessed in serum and MDA and TAS in serum, pancreas, and heart. Pancreatic histology was also evaluated. Sitagliptin in diabetic rats promoted a decrease in glucose, HbA1c, Total-c, and TGs accompanied by a partial prevention of insulinopenia, together, with a decrease in CRP*hs* and IL-1*β*. Sitagliptin also showed a positive impact on lipid peroxidation and hypertension prevention. In conclusion, chronic sitagliptin treatment corrected the glycaemic dysmetabolism, hypertriglyceridaemia, inflammation, and hypertension, reduced the severity of the histopathological lesions of pancreatic endocrine and exocrine tissues, together with a favourable redox status, which might be a further advantage in the management of diabetes and its proatherogenic comorbidities.

## 1. Introduction

Type 2 diabetes mellitus (T2DM) is the most common endocrine disorder worldwide, affecting more than 200 million people [1]. Pathogenesis of this disease involves abnormalities in glucose and lipid metabolism, including inadequate insulin secretion from pancreatic *β*-cells and resistance to insulin activity (insulin resistance) [2]. 

Hyperglycaemia and hyperlipidaemia are the key promoters, through distinct mechanisms, of reactive oxygen species (ROS) and advanced glycation end products (AGEs) production, which causes cell damage and insulin resistance [3, 4]. Moreover, these high levels of glucose and lipids stimulate pro-inflammatory cytokines, promote lipid peroxidation, thus contributing to beta-cell degradation, particularly due to apoptosis pathways [5]. Actually, inflammation and oxidative stress play a major role in type 2 diabetes mellitus (T2DM) pathophysiology, contributing for obesity, insulin resistance and cardiovascular complications, which further aggravate the disease. However, so far, there are no therapeutic options able to efficiently act not only on the glucose control but, and specially, on the prevention of T2DM evolution and its complications, namely, by beta-cell function preservation. 

In T2DM patients, the effect of the glucose-dependent insulinotropic polypeptide (GIP), as well as the secretion of the glucagon-like peptide-1 (GLP-1), is diminished or absent, contributing to insulin secretion deficiency [6]. These two incretins are secreted by the intestine [7] and stimulate insulin secretion by beta-cells, in a glucose-dependent manner [8], preventing hypoglycemia. In animal models, continuous infusion of GLP-1 or injection of long-acting GLP-1 mimetics, such as exendin-4, has shown a remarkable glucose-lowering efficacy, together with an ability to increase beta-cell neogenesis and reduce apoptosis and alpha-cell glucagon secretion [9–11]. Despite the beneficial actions of GLP-1 and GIP, their use as antidiabetic agents (mimetics) is impractical due to their short half-lives, as a result of their rapid inactivation by dipeptidyl peptidase-IV (DPP-IV) [12, 13]. Thus, orally administered DPP-IV inhibitors have emerged as a new class of antihyperglycaemic agents with the ability for extending the biological effects of incretin hormones through the inhibition of their degradation [14, 15], with the advantage of higher stability and bioavailability when compared with the mimetics.

Sitagliptin, an orally available DPP-IV inhibitor developed to be used as a once daily treatment for T2DM, has shown beneficial effects on glycaemic control, reducing HbA1c, and preventing hypoglycemia, as well as on islet mass and function, with no relevant adverse effects [16, 17]. Considering the vast physiological actions promoted by the incretins, not only related with the control of glucose by insulin and glucagon regulation, but also with the peripheral insulin sensitization, cardiac and neuronal protection and beta-cell preservation, the use of an incretin enhancer (such as sitagliptin) might present beneficial effects on diabetes pathophysiology and on prevention of its serious complications, which deserves better elucidation.

The male Zucker Diabetic Fatty (ZDF) rat displays glucose intolerance, marked insulin resistance, and hyperlipidaemia, and becomes overtly diabetic after 8 weeks of age if fed a diet containing 6.5% fat [18]. In the prediabetic state, the male ZDF rat experiences a steady increase in basal insulinaemia and plasma free fatty acid (FFA) levels. Hyperglycemia develops between 8 and 10 weeks of age, leading to overt diabetes and collapsing insulin secretion [19]. This profile mimics the progressive loss of glucose-stimulated insulin secretion in human type 2 diabetes and, thus, the ZDF rat represents a good animal model for studying human T2DM pathophysiology and the effects of therapeutic options [20].

The purpose of this study was, thus, to assess the effects of chronic sitagliptin treatment on the metabolic profile, inflammation, and redox status and pancreas histology in the ZDF rat, an animal model of obese T2DM.

## 2. Material and Methods

### 2.1. Animals and Experimental Design

Male ZDF rats (ZDF/Gmi, *fa/fa*) and their littermates (ZDF/Gmi, +/+) were purchased from Charles River Laboratories (Barcelona, Spain) with 6 weeks of age. Rats were properly housed, handled daily, and kept at a controlled standard temperature (22-23°C), humidity (60%) and light-dark cycles (12/12 hours). Throughout the experiment, the animals were fed distilled water *ad libitum* and rodent maintenance chow (A-04 Panlab, Barcelona, Spain) containing 15.4% of protein and 2.9% of lipids). The chow was adapted to the animal's body weight (BW): 100 mg/g. Animal experiments were conducted according to the European Council Directives on Animal Care and to the National Laws. 

When aged 20 weeks (T0), the diabetic ZDF (*fa/fa*) rats were divided in 2 subgroups (*n* = 8 rats each): a control and a treatment group, receiving, respectively, by oral gavage, once a day (6:00 PM), during 6 weeks, the vehicle (orange juice) and sitagliptin (10 mg/kg/BW/day). The same procedures were adopted with the lean nondiabetic ZDF (+/+) control rats. The ZDF (+/+) control group under sitagliptin treatment showed no relevant differences when compared with the ZDF (+/+) control rats under vehicle and, thus, the results were excluded from tables and figures in order to facilitate data comparison and interpretation.

Food intake and BW were measured each day before treatment and expressed as weekly average values. Systolic blood pressure (SBP), diastolic blood pressure (DBP) and heart rate (HR) were determined in conscious rats using a tail-cuff sphygmomanometer LE 5001 (Letica, Barcelona, Spain) in appropriate restriction cages. Pulse pressure (PP) was calculated by the difference between the systolic and the diastolic readings (PP=SBP−DBP). Blood pressure (BP) values, obtained by averaging 8 to 10 measurements, were recorded by the same person, in a similar peaceful environment. Measurements were performed at T0 and at the end of the study (Tf) with special precautions to minimize stress-induced fluctuations in BP, as previously described [21].

### 2.2. Sample Collection and Preparation

*Blood*: when aged 20 weeks (T0) and at the end of the experience (26 weeks - Tf) the rats were subjected to intraperitoneal anesthesia with a 2 mg/kg BW of a 2:1 (v:v) 50 mg/mL ketamine (Ketalar, Parke-Davis, Lab. Pfeizer Lda, Seixal, Portugal) solution in 2.5% chlorpromazine (Largactil, Rhône-Poulenc Rorer, Lab. Vitória, Amadora, Portugal) and blood samples were immediately collected by venipuncture from the jugular vein into syringes without anticoagulant (for serum samples) or with the appropriate anticoagulant: ethylene-diaminetetraacetic acid (EDTA)-2K for Glycosylated haemoglobin (HbA_1_c) measurement. 

The rats were sacrificed by anesthetic overdose. The pancreas and the heart were immediately removed, placed in ice-cold Krebs' buffer and Bock's fixative, respectively, and carefully cleaned of extraneous fat, lymph nodes and connective tissue. The organs were cross-sectioned and cryopreservated, fixed and processed for paraffin embedding in accordance with subsequent analysis protocols.

### 2.3. Glycaemic and Lipidic Profile Assays

Serum total cholesterol (Total-c) and triglycerides (TGs) were analysed on a Hitachi 717 analyser (Roche Diagnostics) using standard laboratorial methods. Total-c reagents and TGs kit were obtained from bioMérieux (Lyon, France). Serum glucose levels were measured using a Glucose oxidase commercial kit (Sigma, St. Louis, Mo, USA). Considering the variability of serum glucose levels in the rat, glycosylated haemoglobin (HbA_1_c) levels were used as an index of glucose control, through the DCA 2000+ latex immunoagglutination method (Bayer Diagnostics, Barcelona, Spain). Plasma insulin levels were quantified by using a rat insulin Elisa assay kit from Mercodia (Uppsala, Sweden). Insulin sensitivity of individual animals was evaluated using the previously validated homeostasis model assessment (HOMA) index [21]. The formula used was as follows: [HOMA-IR] = fasting serum glucose (mmol/l) x fasting serum insulin (*μ*U/ml)/22.5. The values used (insulin and glucose) were obtained after an overnight of food deprivation.

### 2.4. Inflammatory Profile and Redox Status

Serum levels of interleukin-1*β* (IL-1*β*), tumour necrosis factor *α* (TNF-*α*) and adiponectin were all measured by rat-specific Quantikine ELISA kits from R&D Systems (Minneapolis, USA). High-sensitive C-reactive protein (CRP*hs*) was determined by using a rat-specific Elisa kit from Helica Biosystems Inc. (Fullerton, CA, USA). All assays were performed according to the manufacturers' recommendations, in duplicate.

The thiobarbituric acid reactive-species (TBARs) assay was used to assess serum, pancreas and heart products of lipid peroxidation, via malondialdehyde (MDA), according to that previously described in [22]. Samples were analysed spectrophotometrically at 532 nm using 1,1,3,3-tetramethoxypropane as external standard. The concentration of lipid peroxides (in MDA) was expressed as *μ*mol/l in the plasma and as *μ*mol/g tissue in the pancreas and heart. Ferric reducing antioxidant potential (FRAP) assay was used to estimate serum total antioxidant status (TAS) [23].

### 2.5. Histological Studies

Specimens were paraffin-embedded and the 3 *μ*m thick sections stained for routine histopathological diagnosis with haematoxylin and eosin (HE). All samples were examined by light microscopy using a Microscope Zeiss Mod. Axioplan 2. The degree of injury visible by light microscopy was scored in a single-blind fashion by the pathologist to the animal study group. Endocrine pancreatic damage was assessed by evaluating changes in the islets of Langerhans, namely the shape (architecture), presence of inflammatory infiltrate, fibrosis, vacuolization and intraislets congestion. A semiquantitative rating for each slide ranging from 0 (minimal) to 3 (severe and extensive damage) was assigned to each component. The exocrine pancreatic damage was evaluated, according to the presence of congestion, fibrosis, and inflammatory infiltrate in the interstitial tissues and graded, also, in the same semiquantitative rating.

### 2.6. Statistical Analysis

Results are shown as mean ± standard error of the mean (SEM). The comparison of values between groups was performed by using ANOVA followed by the Bonferroni post hoc test, through appropriate software (GraphPadPrism 5.0 from GraphPad Software Inc., La Jolla, CA, USA). Significance was accepted at a  *P*  less than  .05.

## 3. Results

### 3.1. Effects of Chronic Sitagliptin Treatment on Body Weight and Glycaemic and Lipidic Profiles

Concerning the body weight, no significant differences were encountered between the diabetic and the lean control rats in the beginning of treatments (T0: week 20), despite the obese profile encountered in the diabetic ZDF (*fa/fa*) rats between the 8th and the 14th week (data not shown). At the end of the study (26 weeks), the control diabetic ZDF (*fa/fa*) rats exhibit an 8.7% reduction in their BW (*P* < .001); nevertheless, the lean control group gained weight. Sitagliptin treatment, during 6 weeks, stabilized the loss of weight in the diabetic ZDF *(fa/fa)* rats, even preventing part of the BW loss when compared with the rats without treatment (Table 1).

**Figure 1:**
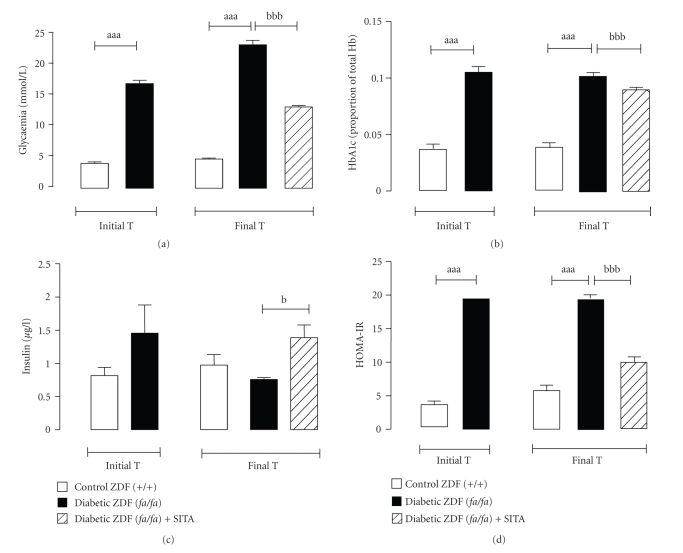
Glycaemic and insulinaemic profiles. Serum Glycaemia (a), HbA1c (b), insulinaemia (c) and insulin resistance (HOMA-IR) index (d), for the control (+/+) and diabetic (*fa/fa*) ZDF rats, in the initial and final times (6 weeks of vehicle or 10 mg/kg BW/day sitagliptin treatment). Comparisons between groups (*n* = 8 each): a - ZDF (*fa/fa*) versus ZDF (+/+) and b - with sita versus without sita; *P* < .05, *P* < .01 and *P* < .001 for one, two or three letters, respectively. HOMA-IR, homeostasis model assessment—insulin resistance.

The determination of serum glucose, HbA1c, Total-c and TGs concentrations was carried at the initial time (T0: 20 weeks old) and at the end of the study (Tf: 26 weeks old). At the T0, the diabetic group showed a hyperglycaemic and a hyperlipidemic profile, also seen at the final time (Figure 1(a) and Table 1). As illustrated in Figure 1(b), the HbA1c values were higher in the diabetic rats than those of the control animals, confirming the glycaemic deregulation. The diabetic ZDF (*fa/fa*) rats have also presented higher levels of Total-c and TGs versus the control ZDF (+/+) animals, in both times (Table 1). 

After 6 weeks of sitagliptin treatment (Tf: 26 weeks), a significant improvement in glycemic control was observed in diabetic ZDF (*fa/fa*) rats (486.3 ± 19.1 mg/dl), when compared with the vehicle-treated diabetic animals (523.3 ± 15.6 mg/dl; *P* < .001) (Figure 1(a)). This pattern of changes is also expressed by the HbA1c levels, which decreased by 11.1% in sitagliptin-treated ZDF (*fa/fa*) rats when compared with the diabetic rats not treated with the drug (Figure 1(b)). TGs were significantly reduced (50%; *P* < .001) in the diabetic rats treated with sitagliptin during 6 weeks versus the diabetic vehicle-treated group (Table 1).

### 3.2. Effects of Chronic Sitagliptin Treatment on Insulin Levels and Insulin Resistance (HOMA-IR)

At the beginning of the study (T0), insulin levels were higher in the diabetic rats than those of the control, but the differences did not reach statistical significance. At the final time, the vehicle-treated ZDF (*fa/fa*) rats exhibit relative insulinopenia (0.75 ± 0.05 *μ*g/l), when compared to vehicle-treated ZDF (+/+) (1.05 ± 0.30 *μ*g/l) (Figure 1(c)), accompanied by a significant augment (*P* < .001) of insulin resistance (HOMA-IR index) (Figure 1(d)). The elevation of insulin resistance was prevented (*P* < .001) in the sitagliptin-treated diabetic (*fa/fa*) rats (Figure 1(d)).

### 3.3. Effects of Chronic Sitagliptin Treatment on Blood Pressure

The vehicle-treated ZDF (*fa/fa*) group showed significantly (*P* < .05) higher levels of systolic and mean BP, together with a trend to higher diastolic and pulse pressure, when compared with the vehicle-treated ZDF (+/+) group. Sitagliptin treatment has significantly prevented the blood pressure rise (hypertension) in the diabetic rats (Table 1).

### 3.4. Effects of Chronic Sitagliptin Treatment on Inflammatory Profile

**Figure 2:**
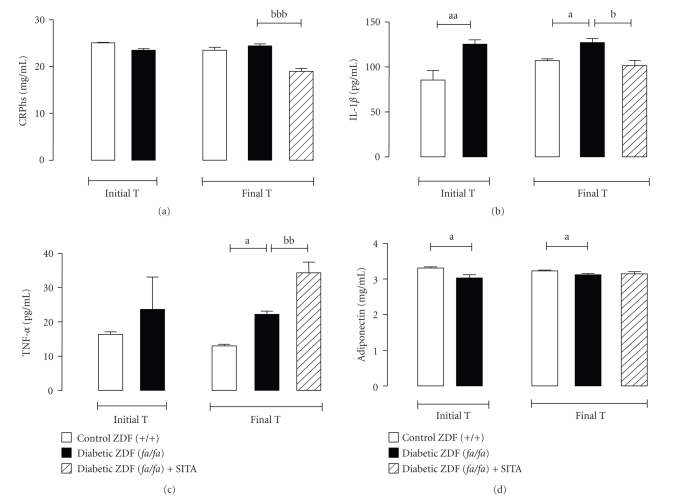
Serum inflammatory markers. Serum CRP*hs *(a), IL-1*β* (b), TNF-*α* (c) and Adiponectin (d) for the control (+/+) and diabetic (*fa/fa*) ZDF rats, in the initial and final times (6 weeks of vehicle or 10 mg/kg BW/day sitagliptin treatment). Comparisons between groups (*n* = 8 each): a - ZDF (*fa/fa*) versus ZDF (+/+) and b - with sita versus without sita; *P* < .05, *P* < .01 and *P* < .001 for one, two or three letters, respectively. CRP*hs*, high-sensitive C-reactive protein; IL-1*β*, interleukin-1beta; TNF-*α*, Tumor necrosis factor-alpha.

Concerning the serum CRP*hs *levels, no significant differences were observed between the diabetic ZDF (*fa/fa*) and the nondiabetic ZDF (+/+) vehicle-treated groups (Figure 2(a)). However, there was higher serum levels of IL-1*β* and TNF-*α* and reduced of adiponectin in the vehicle-treated diabetic ZDF (*fa/fa*) rats when compared with the vehicle-treated nondiabetic (+/+) rats (Figures 2(b), 2(c) and 2(d)). Sitagliptin treatment has significantly decreased the levels of CRP*hs *(*P* < .001) and IL-1*β* (*P* < .05) in the diabetic ZDF rats (Figures 2(a) and 2(b)). However, the diabetic (*fa/fa*) animals under stagliptin therapy showed, at the end of the study, elevated (*P* < .01) levels of TNF-*α* (Figure 2(c)), without significant changes on serum adiponectin contents (Figure 2(d)).

### 3.5. Effects of Chronic Sitagliptin Treatment on Serum and Tissue Redox Status

**Figure 3:**
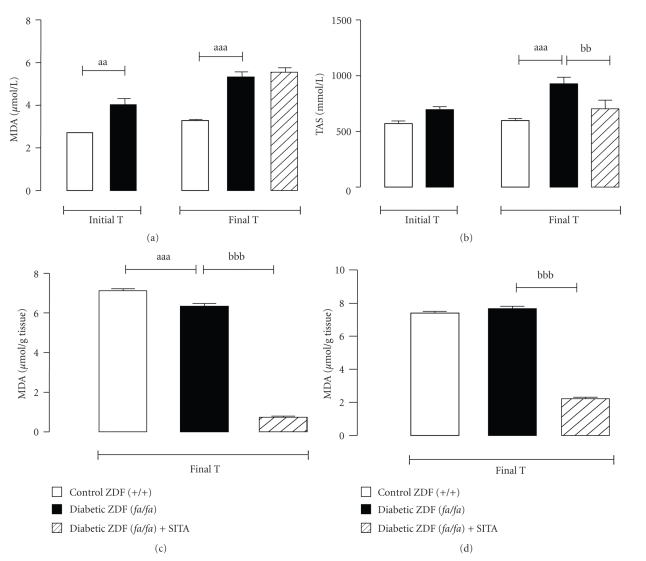
Serum and tissue redox status markers. Serum MDA (a) and TAS (b) and pancreas (c) and heart (d) MDA, for the control (+/+) and diabetic (*fa/fa*) ZDF rats, in the initial and final times (6 weeks of vehicle or 10 mg/kg BW/day sitagliptin treatment). Comparisons between groups (*n* = 8 each): a - ZDF (*fa/fa*) versus ZDF (+/+) and b - with sita versus without sita; *P* < .05, *P* < .01 and *P* < .001 for one, two or three letters, respectively. MDA, malondialdehyde; TAS, total antioxidant status.

The vehicle-treated diabetic ZDF (*fa/fa*) group exhibited significantly higher levels of serum MDA (at the T0 and Tf), accompanied by a compensatory elevation of TAS in the final time (Figures 3(a) and 3(b)). Sitagliptin treatment during 6 weeks has decreased (*P* < .01) serum TAS content, whereas there were no differences in serum MDA levels (Figures 3(a) and 3(b)). On the contrary, we observed a significant reduction of pancreas (*P* < .001) and heart (*P* < .001) MDA levels in the sitagliptin-treated diabetic ZDF (*fa/fa*) rats when compared with the vehicle-treated (*fa/fa*) rats (Figures 3(c) and 3(d)).

### 3.6. Effects of Chronic Sitagliptin Treatment on Pancreatic Histology

**Figure 4:**
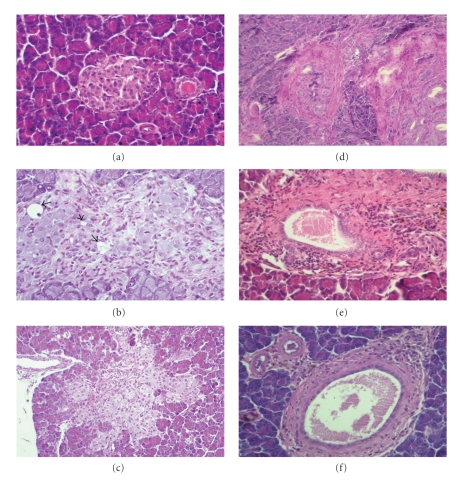
Pancreatic histology at the end of experimental period. Endocrine pancreas (a, b and c): (a) Typical islet from control ZDF (+/+) rats under vehicle treatment, without changes in the endocrine and exocrine pancreas; (b) Extensive fibrosis, vacuolation and loss of architecture in diabetic ZDF (*fa/fa*) rats under vehicle treatment; (c) Diminution in fibrosis intensity and vacuolation in Langerhans islet from diabetic ZDF (*fa/fa*) rats treated for 6 weeks with 10 mg/kg BW/day of sitagliptin, between weeks 20 and 26 (final time); Exocrine pancreas (d, e and f): (d) Severe fibrosis (III) with *neocanaliculi* (original magnification x 200) and (e) Congestion and intense inflammatory infiltrate from diabetic ZDF (*fa/fa*) rats treated with vehicle; (f) Marked decrease in fibrosis severity from diabetic ZDF (*fa/fa*) rats treated with sitagliptin. hematoxylin and eosin staining (original magnification x 400).

In the control rats (ZDF (+/+) under vehicle treatment, there was no pathological changes in the endocrine and exocrine pancreas (Figure 4(a)). Langerhans islets of diabetic ZDF animals treated with sitagliptin presented a diminution in fibrosis intensity (Figure 4(c)). While vehicle-treated diabetic ZDF *(fa/fa)* rats presented a higher number of animals in advanced degrees of fibrosis severity (75.0% of grade 3; 12.5% of grade 2 and of 12.5% grade 1), in the sitagliptin-treated group the severity of fibrosis rating ranged only from 1 to 2 (37.5% and 62.5%, resp) (Figures 4(a) and 4(b)). An amelioration of the inflammatory infiltrate in the endocrine pancreas was encountered when the diabetic ZDF rats were cronically treated with sitagliptin (Table 2). The treated group presented 87.5% rats with grade 1 inflammatory infiltrate, whereas in the vehicle-treated group all rats presented inflammatory infiltrate (37.5% of grade 3 and 62.5% of grade 2). Intra-islet cellular grade 2 vacuolation was present in most of the rats (75%) without treatment (vehicle-treated group). This grade was quantitatively reduced in the treated group, in which only 1 rat (12.5%) presented grade 2 vacuolation, representing the remainder (37.5%) a grade 1 vacuolation. Congestion affected one vehicle-treated diabetic ZDF (*fa/fa*) rat, being completely absent in the sitagliptin group (Table 2). Nevertheless, on the parenchymal structure or islet size, only subtle differences were broadly detected.

All the diabetic ZDF (*fa/fa*) rats without stagliptin treatment exhibited in the exocrine pancreas a variable degree of fibrosis and ductal hypertrophy rating in levels 1, 2 and 3, as shown in Table 2 (Figures 4(d) and 4(e)). All the rats presented inflammatory infiltrate rating from 1 (37.5%) to 2 (62.5%). A grade 2 congestion was observed in most of the vehicle-treated rats (75.0%). Lesions of the exocrine pancreas of diabetic rats chronically treated with sitagliptin, when compared with those without treatment, exhibited a decrease in fibrosis, being absent in most of the animals (62.5%), with the remaining cases showing fibrosis rating in grade 1 and 2 (Figure 4(f)). Despite the presence of grade 1 or 2 (each representing 50%) inflammatory infiltrate in all rats, a reduction in severity was found in one of the animals (Table 2). The severity of congestion suffered a decrease from level 2 to level 1 in 50% of the rats and was completely absent in the other 50% of the group.

## 4. Discussion

Previous reports suggest that local and systemic low-grade inflammation and oxidative stress, which are mainly fuelled by hyperglycaemia and hyperlipidaemia, are important mediators of beta-cell degradation, insulin resistance and T2DM complications in many individuals [24–26]. It is now recognized that adipocytes, particularly those located within the visceral fat, are major secretors of both pro-and antiinflammatory factors, often referred to as adipokines [27, 28]. Several well-known markers of inflammation secreted by the adipose tissue, including IL-6 (which stimulated the hepatic synthesis of CRP), IL-1*β* and TNF-*α*, have been referred as independent predictors of diabetes [28–30]. Adiponectin, an adipokine, has demonstrated antiinflammatory properties, protection against insulin resistance, as well as against the development of atherosclerosis [31–34]. 

In this study, we assessed the effects of chronic sitagliptin treatment on glucose and lipids deregulation and on other cardiometabolic risk factors in an animal model of obese type 2 diabetes mellitus, the ZDF rat. Since the diagnosis of the disease is frequently late, when diabetes pathophysiological mechanisms are already advanced and the complications have already been initiated, we chose to use the diabetic ZDF rats in an established diabetes stage, which, according to our previous data, is when the animals aged 20 weeks [35, 36].

Concerning the ZDF model of type 2 diabetes, our results have demonstrated the key features encountered in type 2 diabetes patients. Therefore, at the beginning of the study (initial time: 20 weeks of age) the diabetic rats presented hyperglycaemia, hypercholesterolaemia, hypertriglyceridaemia, increased HbA1c and hyperinsulinaemia, accompanied by insulin resistance (HOMA-IR). Insulin levels of ZDF (*fa/fa*) rats were already decreased when compared with the controls, indicating an impaired insulin secretion by the pancreatic beta-cell. Furthermore, the ZDF (*fa/fa*) rats presented obesity between the 8th and the 14th week of age (data not shown), but started losing weight until the week 20. This BW decrease continued throughout the experimental period, which might be viewed as a complication of diabetes. Furthermore, the ZDF diabetic rats also presented, when compared with the nondiabetic ZDF (+/+) controls, a pro-inflammatory profile, represented by the reduced content of the antiinflammatory adipokines, adiponectin, and the increased level of the pro-inflammatory cytokines IL-1*β* and TNF-*α*. However, we should identify two surprising aspect encountered in the diabetic ZDF (*fa/fa*) rats at 20 weeks-old, which contrasts with previous data from us concerning the characterization of this animal of obese type 2 diabetes [35, 36], that were related to the almost unchanged serum CRP*hs* levels between the diabetic and the control (nondiabetic) animals and the only slightly (but significantly) lower adiponectin in the ZDF diabetic rats, suggesting that inflammation at this point (week 20) was more closely related with other players (such as TNF-*α* and IL-1*β*) and, as well, that the BW loss (which might represent an pathophysiological aggravation of the disease) might change the pattern of the inflammatory profile. 

At the end of the experience, week 26, the ZDF rats aggravated their diabetic state, viewed by a higher hyperglycaemia, accompanied by increased HbA1c, insulin resistance and reduced plasma concentration of insulin, suggesting that the relative insulinopenic state, which started at the beginning of the study, was aggravated. Moreover, the ZDF diabetic rats continue to lose weight and showed an aggravated hypercholesterolaemia, hypertriglyceridaemia, together with inflammation and hypertension. At this time, however, the increased serum MDA content was accompanied by a compensatory increase in serum TAS, which might explain the unchanged values of tissue (pancreas and heart) MDA between the diabetic and nondiabetic animals. In any case, between the week 20, corresponding to an established diabetes state, and the week 26, the diabetic rats aggravates the disease (viewed mainly by the aggravated hyperglycaemia and the insulinopenia) and its complications (hypertension), which is in agreement with our previous data concerning the metabolic characterization of this model of type 2 diabetes mellitus (the ZDF rat) [35, 36]. 

During the course of the study, the diabetic rats treated once a day with an incretin enhancer, the DDP-IV inhibitor sitagliptin, showed a remarkable beneficial effect on several important parameters, not only those related to the glycaemic control, as should be expected when using an antihyperglycaemic agent, but also on other cardiometabolic perturbations and complications related to diabetes. Therefore, chronic sitagliptin treatment has promoted a reduction of glucose and HbA1c levels, together with a partial correction of insulin reduction and an improvement of insulin resistance (HOMA-IR), which is in agreement with other reports [37, 38]. Furthermore, the reduction of BW was prevented and the hypertriglyceridaemia corrected, which was accompanied by a prevention of diabetes-induced hypertension, as previously suggested by other authors in [38, 39]. Future studies from us will estimate the effects of this DPP-IV inhibitor on the enzyme activity/expression, as well as on levels of GLP-1 and glucagon, in order to have a more detailed picture of how the incretins pathway is affected and its relative contribution for the effects of sitagliptin here reported.

Evaluation of endocrine pancreatic tissue suggests amelioration in Langerhans islets by sitagliptin treatment. In the exocrine pancreas an improvement in sitagliptin-treated rats was also observed. However, results must be carefully interpreted because they superimpose on those lesions presented by diabetic rats without treatment as result of obesity and/or type 2 diabetes. Matveyenko et al. (2009) using HIP rats reported beneficial effects of sitagliptin in endocrine pancreas, together with haemorrhagic pancreatitis in one sitagliptin treated rat, ductal metaplasia in three sitagliptin-treated rats and increased ductal proliferation in all sitagliptin-treated rats, suggesting chronic pancreatitis [40]. Nevertheless, they use a dosage 20 fold larger, with a duration of treatment twice longer than the one used in our present work. Despite the difference in rat specie, dose and route of administration, the studies of Matveyenko et al. (2009), using a DPP IV inhibitor, and of Nachnani et al. (2010) [41], using an injection of GLP-1 agonist to enhance endogenous GLP-1 levels, raise the possibility that the enhancement of endogenous GLP-1 levels could induce undetected low grade asymptomatic chronic pancreatitis. Despite the lower dose used, we observed beneficial effects of sitagliptin on metabolic profile and reduction in inflammatory markers, as well as an amelioration of fibrosis, vacuolization and congestion in endocrine pancreas. Others have observed similar results using FE 999011, an inhibitor of DPP IV, administrated orally in a dose of 10 mg/kg BW once a day [42]. The therapeutic dosage required to improve glucose tolerance, on an acute scale in humans (0.2 mg/kg), is 200-fold lower than the one used in the present study [43]. Our findings suggest that the compensatory change in circulating DPP-IV levels could be avoided by once-daily treatment and/or a lower inhibitor dosage. 

Concerning the markers of inflammation and oxidative stress, this study demonstrated an important effect of sitagliptin on CRP*hs *and IL-1*β* serum levels, reducing the higher levels encountered in the diabetic rats. The effects on these mechanisms have contrasted with those encountered on TNF-*α* and adiponectin, in which an increment and the absence of influence, respectively, were observed, suggesting that distinct mechanisms regulates the different cytokines produced by the adipocyte tissue. The increment on serum TNF-*α* levels might eventually suggest undesirable side effect of sitagliptin. Therefore, it is well known that the inhibition of the serine protease DPP-IV in type 2 diabetes treatment prevents its activation of insulin-releasing peptide hormones. However, DPP-IV also cleaves many other molecules, including chemokines, suggesting that inhibition of this enzyme could have undesired side effects and might be responsible for allergic reactions and runny or stuffy nose, sore throat, and upper respiratory infection, described as sitagliptin side effects [44]. 

The beneficial effect on systemic CRP*hs* and IL-1*β* was accompanied by an improvement of tissue redox status, with a remarkable positive impact on lipid peroxidation in both the pancreas and the heart. These effects, together with a decrease in TGs content, might contribute to reduce pancreatic beta-cell deterioration, which is a feature of diabetes evolution to high deregulated states, and to alleviate the cardiovascular complications that accompany the evolution of the disease and that are responsible for the associated high mortality and morbidity rates worldwide [45]. The blood pressure amelioration found in our study might be secondary to the improvement of glucose and lipidic dysmetabolism, low-grade inflammation and oxidative stress status, which are factors undoubtedly linked with the cardiometabolic complication associated with diabetes. However, a direct favourable influence of sitagliptin on the cardiovascular system might occur, as suggested by the positive impact on heart redox status. Furthermore, the previously suggested antiapoptotic effect of the incretin modulators on the pancreas might be extended to other tissues, such as the heart. This hypothesis should be further reinforced in future studies. An adequate treatment for type 2 diabetes, according to the guidelines, should be focused not only on glycaemia control, but also, on reduction of triglycerides and blood pressure, thus preventing the cardiovascular complications [46–48]. According to previous data, there is yet no sufficient clinical data to assess the real influence of incretin modulators on cardiovascular disease prevention and on long-term cardiovascular safety [49, 50]. 

Several reports have indicated that DPP-IV inhibitors are as antihyperglycaemic as any other oral antidiabetic drugs, with the additional benefit of not promoting hypoglycaemia and weight gain [45]. Further studies, using another antidiabetic agent from other group, should be performed in order to confirm if the beneficial effects now obtained are clearly directly attributed to the mechanism of action of this compound and are not exclusively resulting from the improvement of glycemic control. Since GLP-1 receptors have been identified in several tissues related with the cardiovascular system, such as the cardiomyocytes and vascular endothelial cells, the effects of the incretin-based therapies, such as the DPP-IV inhibitors, point to a potential benefit on attenuation of type-2 diabetes-induced cardiovascular complication [45]. However, the current limitations are related to the lack of log-term clinical studies [49, 50]. In any case, considering the interesting properties demonstrated by these new class of antidiabetic agents, which make them different from the traditional drugs, and if the clinical studies are able to confirm other influences, apart the already reported glycaemic control and HbA1c reduction, in a near future their place in the treatment algorithm might be reviewed. Therefore, if the beneficial effects on beta-cell function preservation, as well as on prevention of diabetic complications, will be further confirmed, they might be recommended not only as adjuvant therapy when other antidiabetics fail to control glycaemia and HbA1c levels, but also, as one of the main choices for type 2 diabetes management and prevention of complications.

## 5. Conclusions

This study, using a model of obese T2DM (the ZDF rat), demonstrated that chronic inhibition of DPP-IV by sitagliptin can correct the glycaemic dysmetabolism, hypertriglyceridaemia, inflammation and hypertension, reduce severity of histopathological lesions of endocrine and exocrine pancreas, jointly, with a favourable influence on the pancreas and heart lipid peroxidation, which have been identified as the key pathophysiological mechanism underlying insulin resistance, beta-cell degradation and associated micro-and-macrovascular complications. These influences here reported may become further advantages in the therapeutics of type 2 diabetes and in the prevention/management of its pro-atherogenic macrovascular complications.

## Figures and Tables

**Table 1:** Body weight, lipid profile and blood pressure in the control and diabetic ZDF rats at the initial and final time (6 weeks of vehicle or sitagliptin treatment).

	Initial Time (20 wks)		Final Time (26 wks)		

	Control ZDF (+/+)	Diabetic ZDF (*fa/fa*)	Control ZDF (+/+)	Diabetic ZDF (*fa/fa*)	

Groups	(*n* = 16)	(*n* = 16)	Vehicle (*n* = 8)	Vehicle (*n* = 8)	Sitagliptin (*n* = 8)
BW (g)	406.70 ± 6.83	388.10 ± 8.87	445.70 ± 8.16	354.40 ± 8.85^aaa^	380.00±14.46
Total-c (mg/dl)	77.50 ± 1.50	155.50 ± 3.50^aaa^	93.00 ± 2.96	193.00 ± 9.79^aaa^	193.10±4.62
TGs (mg/dl)	115.00 ± 11.00	374.50 ± 4.95^a^	154.00 ± 19.14	400.20 ± 27.00^aaa^	237.10 ± 22.54^bbb^
Systolic (mmHg)	115.50 ± 0.83	125.20 ± 0.27	116.00 ± 2.52	127.80 ± 1.23^a^	101.60 ± 0.78^bbb^
Diastolic (mmHg)	100.98 ± 0.82	91.46 ± 0.83	103.50 ± 1.94	112.70 ± 3.98	94.86 ± 0.70^bbb^
Mean (mmHg)	104.25 ± 0.25	108.20 ± 1.42	104.30 ± 4.25	117.40 ± 3.04^a^	96.86 ± 0.51^bbb^
Pulse P (mmHg)	14.52 ± 0.98	33.74 ± 0.37	14.00 ± 4.16	15.09 ± 3.08	6.71 ± 1.11^b^

BW, body weight; P, pressure; SITA, sitagliptin; Total-c, Total-cholesterol; TGs, triglycerides; ZDF, Zucker diabetic fatty. Values are means ± SEM of *n* rats. Comparisons between groups: a - ZDF (*fa/fa*) versus ZDF (+/+) and b - sitagliptin versus vehicle; *P* < .05, *P* < .01 and *P* < .001 for one, two or three letters, respectively.

**Table 2:** Number of rats exhibiting the different pathology scores observed in endocrine (A) and exocrine (B) pancreas.

A-Endocrine pancreas lesions																

Evaluated	Inflammatory				Fibrosis				Intra islet				Congestion			
parameters	Infiltrate								Vacuolation							
Score	0	1	2	3	0	1	2	3	0	1	2	3	0	1	2	3

Groups (*n* = 8, each)																

ZDF (+/+) vehicle	7	1	0	0	6	2	0	0	8	0	0	0	8	0	0	0
ZDF (*fa/fa*) vehicle	0	0	5	3	0	1	1	6	2	0	6	0	7	0	1	0
ZDF (*fa/fa*) sitagliptin	1	7	0	0	0	5	3	0	4	3	1	0	8	0	0	0

B – Exocrine pancreas lesions																

Evaluated	Inflammatory						Fibrosis						Congestion			
parameters	Infiltrate															
Score	0	1	2	3			0	1	2	3			0	1	2	3

Groups (*n* = 8, each)																

ZDF (+/+) vehicle	8	0	0	0			7	1	0	0			8	0	0	0
ZDF (*fa/fa*) vehicle	0	3	5	0			0	3	3	2			2	6	0	0
ZDF (*fa/fa*) sitagliptin	0	4	4	0			6	1	1	0			4	4	0	0

ZDF, Zucker diabetic fatty.
